# Comparison of Crotalidae Immune F(ab’)2 (Equine) and Crotalidae Polyvalent Immune Fab (Ovine) at Establishing Control of Initial Envenomation Symptoms in Louisiana *Agkistrodon* Envenomations

**DOI:** 10.1007/s13181-025-01093-6

**Published:** 2025-09-02

**Authors:** Taylor Sanders, Christine Murphy, Greggory Davis, Christina Aspinwall, Lauren Theriot, Christopher McVicker, Thomas Arnold

**Affiliations:** 1 Emergency Medicine Residency Program, Louisiana State University Health Sciences Center School of Medicine - New Orleans, Baton Rouge Branch Campus, 5246 Brittany Drive, MEIC Building, 3rd Floor, Baton Rouge, LA 70808 USA; 2 Department of Emergency Medicine, Division of Medical Toxicology, Atrium Health’s Carolinas Medical Center, 1000 Blythe Blvd., Charlotte, NC 28203 USA; 3 Our Lady of the Lake Regional Medical Center, 5000 Hennessy Blvd., Baton Rouge, LA 70808 USA; 4 Department of Emergency Medicine, LSU Health Shreveport, 1501 Kings Highway, PO Box 33932, Shreveport, LA 71130-3932 USA; 5 Louisiana Poison Center, Shreveport, LA USA

**Keywords:** Agkistrodon envenomation, Copperhead envenomation, Cottonmouth envenomation, Antivenom, Snakebite

## Abstract

**Introduction:**

Copperheads and cottonmouths are responsible for most snake envenomations in Louisiana. While the United States Food and Drug Administration has approved both Crotalidae polyvalent immune Fab (FabAV) and Crotalidae immune F(ab’)_2_ (Fab2AV) for *Agkistrodon* envenomations, data is limited comparing their efficacies for this indication.

**Methods:**

This is a retrospective study comparing FabAV to Fab2AV in the treatment of suspected *Agkistrodon* envenomations in Louisiana between April 2017 and October 2024. Cases identifying rattlesnakes were excluded. The primary outcome was the need for additional antivenom doses to achieve control after the initial antivenom dose.

**Results:**

One hundred fifty-eight patients received FabAV or Fab2AV, with 100 cases meeting inclusion criteria. Fifty-seven patients received FabAV and 43 received Fab2AV. The snake was identified as copperhead in 48 cases, cottonmouth in 23, and unidentified in 29. In the FabAV cohort, the initial number of vials ranged from four to 12 with a median dose of four vials. Nine FabAV cases (16%) required additional vials for initial control after the first dose. In the Fab2AV cohort, all patients received 10 vials initially, with 24 cases (56%) requiring additional vials for initial control after the first dose. There was a significant difference in the percentage of patients requiring additional control vials between FabAV and Fab2AV.

**Conclusion:**

In this cohort of suspected *Agkistrodon* envenomations, control with initial recommended dosing was more often achieved with FabAV compared to Fab2AV (84% vs. 44%). The results suggest potential benefit to hospitals stocking FabAV in Louisiana and possibly other *Agkistrodon-*predominant regions.

## Introduction

Envenomations by snakes native to the United States (U.S.) can invoke significant morbidity and cost to those afflicted. The majority of these envenomations (98%) reported to U.S. poison centers are due to species within the subfamily *Crotalinae*, known as pit vipers (crotalines) [1]. Pit vipers native to the U.S. include numerous rattlesnake species (*Crotalus* and *Sistrurus* genera) and four *Agkistrodon* species, two copperheads (*A. contortrix*,* A. laticinctus*) and two cottonmouths (*A. piscivorus*,* A. conanti*). Specifically, *Agkistrodon* species account for the majority of envenomations in the U.S., constituting 77% of envenomation calls to U.S. poison centers due to identified native pit vipers in 2023 [1]. This holds true for the state of Louisiana, a natural habitat for one copperhead (*A. contortrix*) and one cottonmouth (*A. piscivorus*) species. According to internal data from a ten-year Louisiana Poison Center review, 82% of human snake envenomations with snake identification involved an *Agkistrodon* species, 53% copperheads and 29% cottonmouths.

Polyvalent antivenoms have been the benchmark for treatment of pit viper envenomations in the U.S. since Wyeth Pharmaceuticals released its equine whole IgG product, Antivenin *Crotalidae* Polyvalent, in 1953. In 2000, the U.S. Food and Drug Administration (FDA) approved *Crotalidae* polyvalent immune fab [ovine] (BTG International Inc) under the brand CroFab^®^ (FabAV) for the treatment of native pit vipers. As a single Fab component of sheep IgG (devoid of the more immunogenic Fc portion), FabAV has been associated with fewer adverse reactions compared to whole IgG antivenom [2, 3]. Venom from four native U.S. pit viper species (*C. atrox*, *C. adamanteus*, *C. scutulatus*, *A. piscivorus*) is used in the FabAV manufacturing process. Preclinical research showed strong potency of FabAV for the prevention of lethality against venoms of two *Agkistrodon* species (*A. piscivorus*, *A. contortrix*) in a murine model (on par with rattlesnake venom neutralization) [4]. There is also randomized, double-blind, placebo-controlled evidence of its efficacy improving limb function recovery in copperhead (*A. contortrix*) envenomations [5]. The recommended dosing of FabAV is four to six vials as needed to achieve control (control vials), which can be increased up to twelve vials in life-threatening envenomations [6, 7]. This dosing recommendation is based on postmarketing data showing higher median doses needed to achieve control in severe envenomations [8]. Once control is achieved, the manufacturer recommends two additional vials (maintenance vials) every six hours for 18 h. The maintenance vials are manufacturer recommended based on two premarketing clinical trials [9, 10] showing high recurrence rates of venom effects after control was achieved with initial dosing. However, with data suggesting as-needed dosing is superior to scheduled maintenance dosing, FabAV maintenance vials are not universally recommended by toxicologists for rattlesnake envenomations [11], nor are they utilized by toxicologists in the majority of cases involving copperheads [12].

*Crotalidae* immune F(ab’)2 (Instituto Bioclon) became available in 2018 under the brand name Anavip^®^ (Fab2AV) with FDA approval to treat North American rattlesnake envenomations. Venom from two non-U.S. native pit viper species is used in the Fab2AV manufacturing process (*Crotalus simus and Bothrops asper*). FDA approval for this product was expanded in 2021 to include U.S. *Agkistrodon* species after a post-hoc analysis of 21 copperhead envenomations from the original clinical trial [13] showed non-inferiority of Fab2AV for the initial control of copperhead envenomations compared to FabAV [14]. The sample size was too small to determine a statistically significant comparison of the two antivenoms. The FDA approved the expanded indication considering this retrospective analysis along with murine data showing Fab2AV neutralization of *Agkistrodon* venom and protection against lethality [15]. The recommended dosing of Fab2AV is an initial dose of ten vials, with additional vials only recommended if needed to achieve control or reestablish control for the recurrence of symptoms [16]. Routine maintenance vials are not part of the standard dosing as Fab2AV has a longer half-life, 133 h [16], compared to FabAV, 15 h [6], and is more efficacious at preventing delayed coagulopathy [13].

With FabAV and Fab2AV both available, U.S. hospitals now have two FDA approved antivenom options to treat native pit viper envenomations. In line with other hospitals, a Louisiana hospital system switched its formulary snake antivenom from FabAV to Fab2AV in 2021. This substitution was in consideration of the expanded FDA approval of Fab2AV to include treatment of *Agkistrodon* envenomations and its lower acquisition cost per vial. With *Agkistrodon* species being responsible for the majority of snake envenomations in this hospital system’s service area, the aim of this study was to compare the efficacy of FabAV to Fab2AV at controlling venom effects in suspected *Agkistrodon* envenomations.

## Methods

### Design and Setting

This is a retrospective observational study of patients presenting to a Louisiana hospital system with suspected *Agkistrodon* envenomation. Patients treated with either FabAV or Fab2AV between April 2017 and October 2024 were included. The study was approved by the system institutional review board with a waiver of patient consent. The system is comprised of multiple urban and rural hospitals with varying levels of service. Individual hospitals within the system began adopting the electronic medical record (EMR) utilized in April 2017. The system formulary switched from FabAV to Fab2AV in 2021. Hospitals began administering Fab2AV once their local supply of FabAV was exhausted.

### Selection of Participants

The system’s EMR was queried for all administrations of FabAV and Fab2AV during the study period, and associated patient encounters were reviewed. Patients with a documented snake bite and local signs of envenomation, specifically swelling or ecchymosis, were included. The species of snake was determined by identification documented in the EMR or photograph if available. Cases were excluded if the snake was identified as a rattlesnake, the patient received both antivenoms, the initial antivenom dose was less than four vials FabAV or 10 vials Fab2AV, the initial antivenom dose was administered greater than 24 h after envenomation, the patient was transferred to an outside facility, or records were unobtainable.

### Data Collection

For included cases, the EMR was reviewed by one of two reviewers. Patient demographics, bite location, antivenom, and hematologic parameters (platelet count, prothrombin time (PT), fibrinogen) were extracted from the EMR. Additionally, reviewers collected data on snake type, circumstances surrounding the bite, initial number of antivenom vials given, whether additional doses of antivenom were needed for initial control, total number of vials administered (control vials plus reemergence and/or maintenance vials), adverse reactions, presence of systemic signs of envenomation, surgical interventions, and envenomation severity.

Snake types included copperheads (*A. contortrix*), cottonmouths (*A. piscivorus*), and unidentified crotalines. Systemic signs were defined as hypotension (systolic blood pressure less < 90 mmHg or mean arterial pressure < 65 mmHg), syncope, vomiting, or diarrhea. Envenomations were categorized as mild if swelling or tenderness was documented as crossing zero to one major joints (wrist, elbow, ankle, knee) and moderate if swelling or tenderness was documented as crossing two major joints [5]. Envenomations were categorized as severe for documentation of any of the following: swelling to an entire extremity (crossing the shoulder or hip), systemic signs, or severe hematologic effects as determined by the authors (platelets < 100,000/mm^3^, PT > 20 s, fibrinogen < 100 mg/dL, or bleeding away from bite site).

### Outcomes

The primary outcome of the study was to compare the proportion of cases in the FabAV group to the Fab2AV group requiring additional antivenom doses for initial control of venom effects after initial dosing. Evidence of control was defined as documentation of arrest of progression of swelling or tenderness, stabilization of hematologic parameters if abnormal, and resolution of any systemic signs [10]. Initial doses were categorized as *control vials*, as well as any additional doses administered prior to the above criteria being met.

Secondary outcomes included the total number of vials administered per case and the occurrences of adverse reactions to the antivenoms. *Total vials* included control vials plus any additional vials administered beyond the amount needed for initial control, whether for maintenance or for documentation of reemergence after control had been established. Additional doses were categorized as *maintenance vials* if there was no documentation of progression, worsening, or reemergence of swelling or tenderness prior to administration, hematologic parameters were stabilized, systemic signs were absent or resolved, and the doses were ordered as an order set to be administered after a set time. Adverse reactions were included for documentation of any of the following occurring after antivenom was initiated: allergic reaction, hypotension, throat swelling, difficulty swallowing, rash, edema, or respiratory signs or symptoms.

### Analysis

Study data were managed using our facility hosted Research Electronic Data Capture tools (REDCap) [17, 18]. After applying exclusion criteria, 10% of the remaining 100 charts (*n* = 10) were independently reviewed by both reviewers. A kappa statistic was calculated for variables requiring clinical interpretation (e.g., severity) to assess interrater reliability. All categorical data are presented as counts with associated percentages and proportions were compared with a chi square test. When expected cell counts were less than 5, Fisher’s Exact test was used instead. Continuous variables are presented as the medians with 25th and 75th interquartile range (IQR) for data with non-normal distributions. Differences between groups for continuous variables were compared with a Wilcoxon rank sum test. Initial vials, control vials, total vials, and percentage of cases requiring additional doses for control were compared between all *Agkistrodons* and unidentified crotalines as well as between the two *Agkistrodon* species. For all statistical tests, a two-sided p-value of < 0.05 was considered statistically significant. Data were analyzed using statistical software R (version 4.4.2, R Foundation for Statistical Computing, Vienna, Austria) [R Core Team].

## Results

### Baseline Characteristics

**Fig. 1:**
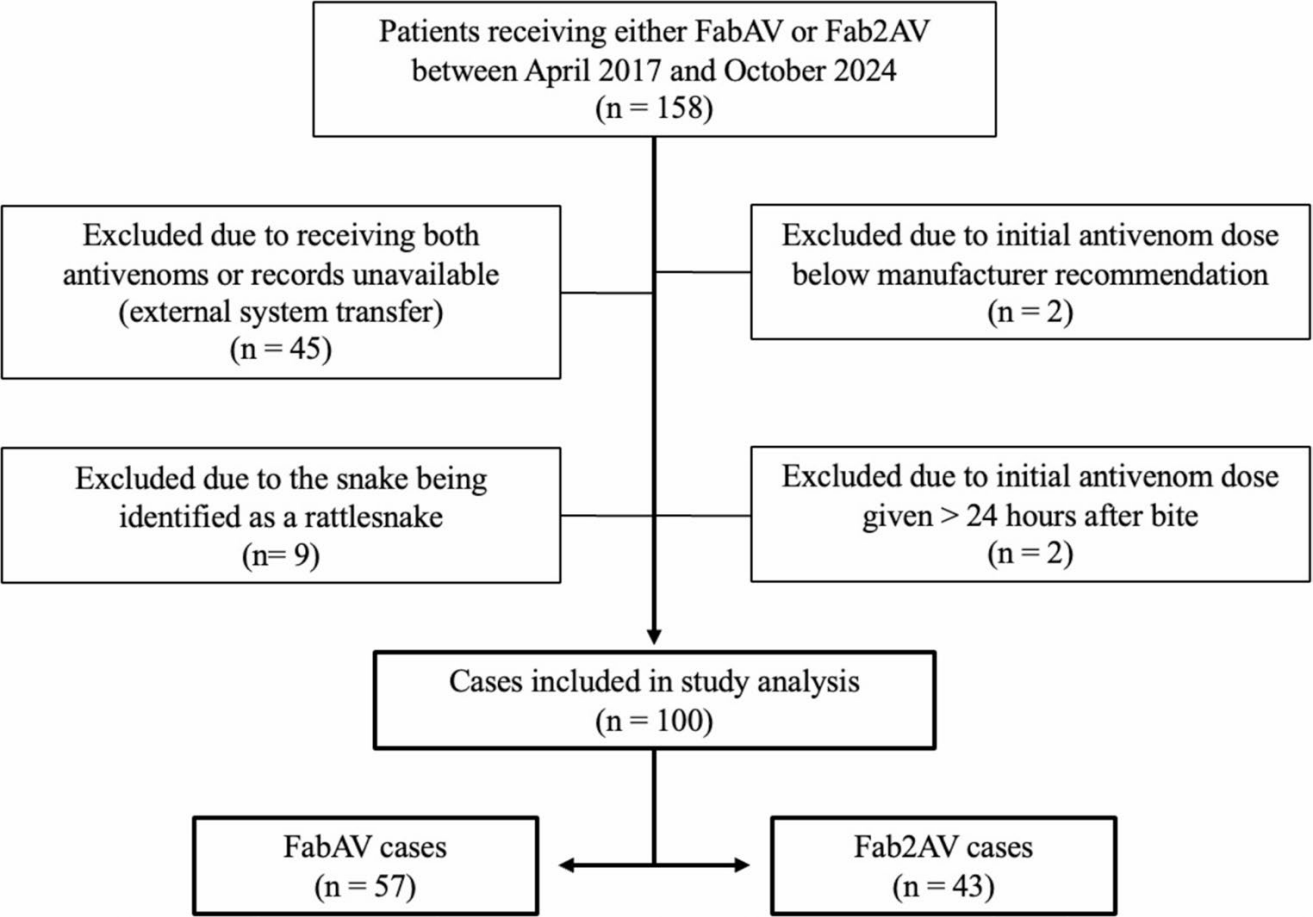
Study inclusion flow diagram

One hundred fifty-eight patients receiving either FabAV or Fab2AV for documented snake envenomation between April 2017 and October 2024 were identified in the EMR. Fifty-eight cases were excluded (Fig. 1). The majority of exclusions (78%) were due to the patient being transferred to or from an outside facility where they either received the alternate antivenom or records were unavailable. Nine cases were excluded due to identified rattlesnake envenomation.

Of the 100 analyzed cases, 57 patients received FabAV (57%) and 43 patients received Fab2AV (43%). Copperheads were identified in 48 of the 100 analyzed cases, and cottonmouths in 23. Twenty-nine analyzed cases were clinically consistent with crotaline envenomation, however the snake species remained unidentified. Fourteen percent (27% of hand bites) were categorized as provoked bites with documentation of the patient, all male, purposefully reaching for the snake. The majority of envenomations were classified as mild (81%), but there were four severe cases, all in copperhead bites to the hand. Two patients were hypotensive (one with vomiting and one with syncope) prior to antivenom administration and did not require vasopressors. The other severe cases were due to swelling of the entire arm (one was also associated with vomiting). Three of the 48 patients with hand bites and one with a foot bite underwent surgical debridement for concern of “compartment syndrome” without measurement of compartment pressures. There were no readmissions to the hospital system within 30 days of discharge. Interrater reliability between chart reviews of 10% of cases (*n* = 10) was perfect (kappa = 1.0). Characteristics of the study sample are outlined in Table 1.

**Table 1:** Baseline characteristics of all included patients

Characteristic	Overall *n* = 100	Copperhead *n* = 48	Cottonmouth *n* = 23	Unidentified *n* = 29
Male (%)	70 (70%)	35 (73%)	18 (78%)	17 (59%)
Median Age (Q1, Q3)	31 (13, 54)	36 (15, 34)	35 (12, 54)	16 (10, 48)
Provoked	14 (14%)	9 (19%)	5 (22%)	0 (0%)
Surgical debridement	4 (4%)	2 (4.7%)	1 (4.5%)	1 (3.4%)
Bite Location				
Hand	48 (48%)	30 (63%)	14 (61%)	4 (14%)
Arm proximal to hand	4 (4%)	3 (6.3%)	0 (0%)	1 (3.4%)
Foot	34 (34%)	10 (21%)	9 (39%)	15 (52%)
Leg proximal to foot	13 (13%)	4 (8.3%)	0 (0%)	9 (31%)
Trunk	1 (1%)	1 (2.1%)	0 (0%)	0 (0%)
Head/neck	0 (0%)	0 (0%)	0 (0%)	0 (0%)
Severity				
Mild	81 (81%)	37 (77%)	20 (87%)	24 (83%)
Moderate	15 (15%)	7 (15%)	3 (13%)	5 (17%)
Severe	4 (4%)	4 (8.3%)	0 (0%)	0 (0%)

Unless otherwise specified, values are n (%)

Hemotoxicity in copperhead envenomations has previously been defined as platelets < 140,000/mm^3^, fibrinogen < 170 mg/dL, PT > 15 s, and aPTT > 36 s [19, 20]. One copperhead envenomation in this study was associated with thrombocytopenia (nadir platelet count of 132,000/mm^3^) and one cottonmouth envenomation was associated with hypofibrinogenemia (fibrinogen 165 mg/dL). No patients had evidence of severe hemotoxicity as defined in the methods and there was no documentation of antivenom administrations for treatment of hematologic abnormalities.

### Additional Doses of FabAV Needed for Control

The median initial FabAV dose was four vials (IQR: 4–6) with an initial dose of 12 vials in one severe case. After initial dosing, additional vials were needed for initial control in nine of 57 (16%) cases receiving FabAV (Table 2). This included five of 37 (14%) cases identifying one of the two *Agkistrodon* species. Of these *Agkistrodon* cases, two of 25 (8%) copperhead envenomations and three of 12 (25%) cottonmouth envenomations required additional control vials (Table 3).

**Table 2:** Comparison of FabAV and Fab2AV requirements for initial control of envenomation symptoms and total doses administered

Antivenom	All cases			Agkistrodon species			Unidentified		
	FabAV *n* = 57^a^	Fab2AV *n* = 43^a^	*p*-value ^b^	FabAV *n* = 37^a^	Fab2AV *n* = 34^a^	*p*-value ^b^	FabAV *n* = 20^a^	Fab2AV *n* = 9^a^	*p*-value ^c^
Initial Dose (Vials)	4 (4, 6)	10 (10, 10)		4 (4, 6)	10 (10, 10)		4 (4, 6)	10 (10, 10)	
Cases Needing Additional Doses for Initial Control	9 (16%)	24 (56%)	< 0.001*	5 (14%)	16 (47%)	0.002*	4 (20%)	8 (89%)	< 0.001*
Total Vials	10 (6, 12)	18 (10, 20)		10 (4, 12)	14 (10, 20)		10 (8, 12)	20 (20, 24)	

^a^ n (%), Median (Q1, Q3); ^b^ Pearson’s Chi Squared test, ^c^ Fisher’s Exact test; * Statistically significant

**Table 3:** Comparison of FabAV and Fab2AV requirements for initial control of envenomation symptoms and total doses administered

Antivenom	Copperheads			Cottonmouths		
	FabAV *n* = 25^a^	Fab2AV *n* = 23^a^	*p*-value ^b^	FabAV *n* = 12^a^	Fab2AV *n* = 11^a^	*p*-value ^b^
Initial Dose (Vials)	4 (4, 6)	10 (10, 10)		4 (4, 5)	10 (10, 10)	
Cases Needing Additional Doses for Initial Control	2 (8%)	14 (61%)	< 0.001*	3 (25%)	2 (18%)	> 0.9
Total Vials	10 (4, 12)	18 (10, 20)		10 (4, 12)	10 (10, 20)	

^a^ n (%), Median (Q1, Q3); ^b^ Fisher’s Exact test; * Statistically significant

### Additional Doses of Fab2AV Needed for Control

In the Fab2AV cohort, every patient received 10 vials initially. Additional vials for initial control were required in 24 of 43 (56%) cases, including 16 of 34 (47%) cases identifying one of the two *Agkistrodon* species (Table 2). The *Agkistrodon* cases requiring additional control doses of Fab2AV included 14 of 23 (61%) copperhead envenomations and two of 11 (18%) cottonmouth envenomations (Table 3). The need for additional doses of antivenom to achieve control after the initial dose occurred more frequently in the Fab2AV cohort compared to the FabAV cohort when comparing patients envenomated by copperheads (*p* < 0.001), all *Agkistrodons* (*p* = 0.002), and all unidentified crotalines (*p* < 0.001). The difference between FabAV vs. Fab2AV for the need of additional control vials for cottonmouths was not statistically significant (Tables 2 and 3).

### Additional and Total Vials

In the FabAV cohort, patients received additional vials, beyond the amount needed for initial control, in 38 of 57 cases (67%). All 38 cases received maintenance vials, including one mild copperhead envenomation to the foot having also received six vials for documentation of reemergence between maintenance doses. The median number of total FabAV vials administered per case was 10 (IQR: 6–12). In the Fab2AV cohort, patients received additional vials, beyond the amount needed for initial control, in nine of 43 cases (21%). Two of these cases had documentation of reemergence after control was achieved with the initial dose. The median number of total Fab2AV vials administered per case was 18 (IQR: 10–20) (Tables 2 and 3).

### Comparison after Exclusion of Cases Receiving Additional Doses

A post-hoc analysis was performed of the 53 cases not receiving additional doses after initial control was obtained, with all vials being categorized as control vials (Table 4). The median initial dose was four vials (IQR: 4–6) for FabAV and all Fab2AV cases received 10 vials initially. Additional control vials were required in two of 19 (11%) FabAV cases and 18 of 34 (53%) Fab2AV cases. The median total FabAV dose was four vials per case (IQR: 4–6) and the median total Fab2AV dose was 14 vials per case (IQR: 10–20). Differences in bite severity and location between groups did not reach statistical significance. The need for additional doses of antivenom to achieve control after the initial dose occurred more frequently in the Fab2AV cohort compared to the FabAV cohort in all cases, those involving identification of an *Agkistrodon* species, and those specifically identifying a copperhead. The difference in the need for additional control vials with FabAV vs. Fab2AV did not reach statistical significance in the 11 cases involving an unidentified crotaline (20% vs. 83%, *p* = 0.08) and the 13 cases identifying a cottonmouth (20% vs. 13%, *p* > 0.9).

**Table 4:** Comparison of FabAV and Fab2AV requirements for initial control of envenomation symptoms in cases not receiving additional vials after initial control was obtained

		All cases			Agkistrodon species			Copperheads		
Antivenom		FabAV *n* = 19^a^	Fab2AV *n* = 34^a^	*p*-value ^b^	FabAV *n* = 14^a^	Fab2AV *n* = 28^a^	*p*-value ^b^	FabAV *n* = 9^a^	Fab2AV *n* = 20^a^	*p*-value ^b^
Initial Dose (Vials)		4 (4, 6)	10 (10, 10)		4 (4, 4)	10 (10, 10)		4 (4, 4)	10 (10, 10)	
Cases Needing Additional Doses for Initial Control		2 (11%)	18 (53%)	0.002*	1 (7%)	13 (46%)	0.015*	0	12 (60%)	0.003*
Control/Total Vials		4 (4, 6)	14 (10, 20)		4 (4, 4)	10 (10, 20)		4 (4, 4)	15 (10, 20)	
**Severity**	Mild Moderate Severe	18 (95%) 0 1 (5%)	28 (82%) 5 (15%) 1 (3%)	0.3	13 (93%) 0 1 (7%)	24 (86%) 3 (11%) 1 (4%)	0.6	8 (89%) 0 1 (11%)	16 (80%) 3 (15%) 1 (5%)	0.6

^a^ n (%), Median (Q1, Q3); ^b^ Fisher’s Exact test; * Statistically significant

### Adverse Reactions

There were four adverse reactions to antivenom administration with three in the FabAV cohort (5.3%) and one in the Fab2AV cohort (2.3%) (Table 5). This difference did not meet statistical significance (*p* = 0.6).

**Table 5:** Description of adverse reactions to FabAV and Fab2AV

Antivenom	Signs and Symptoms	Interventions	Course
FabAV	Flushing Eyelid swelling	Epinephrine IM Diphenhydramine IV Methylprednisolone IV	Dose held then restarted at slower rate. Additional vials given later without issue.
FabAV	Subjective throat swelling Difficulty swelling	Diphenhydramine IV Methylprednisolone IV	Occurred after completion of dose. Did not reoccur.
FabAV	Erythematous rash to arm proximal to infusion	N/a	Dose continued at current rate. Resolved after completion of infusion. Did not reoccur.
Fab2AV	Subjective throat swelling Difficulty swelling	Diphenhydramine IV Methylprednisolone IV	Dose continued at current rate and completed. Did not reoccur.

## Discussion

In this population of pit viper envenomations suspected to be due to *Agkistrodons*, initial control was more often attained with the first dose of FabAV compared to Fab2AV. This difference was significant when the snake was an unidentified crotaline, identified as a copperhead, and in the total cases identifying one of two *Agkistrodon* species. There were no differences in group characteristics with the exception of more provoked bites in the FabAV cohort. Envenomation severities were comparable between groups. While one case of thrombocytopenia and one case of hypofibrinogenemia did occur, there were no occurrences of severe hemotoxic effects as defined in the methods. This is akin to none being seen in copperhead envenomations when coagulation parameters were compared to envenomations by other crotalines [21]. Adverse reactions occurred with both antivenoms and involved mostly subjective symptoms, however one patient receiving FabAV developed visible swelling to their eyelid requiring one dose of epinephrine. All other cases resolved quickly with antihistamines and corticosteroids and no cases resulted in abandonment of antivenom treatment.

With the significant difference in additional, non-control vials between groups, a post-hoc analysis was performed excluding cases receiving any additional vials beyond the amount needed for initial control. In a review of U.S. snake envenomations managed by toxicologists, maintenance dosing was not used in the majority of FabAV cases, including those involving copperheads [12]. Additionally, seven Fab2AV cases in the current study received maintenance vials, possibly due to the providers’ confounding of recommended dosing for FabAV with Fab2AV after the formulary transition. Regardless of antivenom, these maintenance vials may have eliminated the need for additional control vials by preventing progression of local effects, thus acting as control vials and altering the results. Cases with reemergence vials were also excluded to account for the potential increased frequency of evaluations for loss of control in cases not receiving maintenance vials. When excluding the cases with additional vials, initial control was still more often attained with the first (only) dose of FabAV compared to Fab2AV. This difference was statistically significant in total cases of suspected *Agkistrodon* envenomation, those with one of the two *Agkistrodon* species being identified, and those specifically identifying copperheads. The difference did not meet statistical significance when the snake was unidentified or identified as a cottonmouth, although comparison sets were small.

This study’s findings are similar to a comparison of FabAV and Fab2AV for the treatment of copperhead envenomations in Texas [22]. That study only included bites due to *A. contortrix*, which is the only copperhead native to Louisiana and the snake identified most often in the present study. Many explanations could reconcile the mechanism underlying enhanced efficacy of FabAV for control of U.S. *Agkistrodon* envenomations. As pointed out by Greene and Teshon [22], single Fab fragment antivenoms may penetrate tissues more efficiently than Fab2 fragment antivenoms, making them more suitable to neutralize deposited venom proteins and halting local tissue effects. Without discounting the pharmacokinetic and pharmacodynamic distinctions, another possibility, with evidence from premarketing trials, is that FabAV is developed using *Agkistrodon*-specific antibodies while Fab2AV is not. Efficacy in mice does not prove equivalent efficacy in humans; however, there are 5.1 times the minimum mouse LD_50_ neutralizing units for *A. piscivorus* venom in four vials of FabAV compared to 10 vials of Fab2AV, according to their package inserts [6, 16]. In one study referenced in the FDA package insert of Fab2AV, Sanchez, et al. stated FabAV “was considerably more effective in neutralizing the venoms of *A. p. leucostoma* (now considered the same species as *A. piscivorus*), *A. c. contortrix* and *A. c. laticinctus*” compared to Fab2AV [15]. In that study, FabAV had 3.5 and 3.3 times the neutralization potency (defined as the ED_50_ or the dose required to neutralize three times the LD_50_ in mice) for *A. contortrix* and *A. piscivorus* venom, respectively, compared to Fab2AV. Interestingly, after removing cases with potential interference from additional vials, the median dose of Fab2AV needed for control in the current study was 3.5 times greater than the median dose of FabAV.

Hospitals are tasked with stocking medications that are most beneficial to their patients while also maintaining financial stewardship. This is often influenced by the wholesale drug prices available, which for this hospital system is 1.86 times greater for FabAV than Fab2AV, per vial. Previous studies have compared costs between FabAV and Fab2AV for the treatment of U.S. snake envenomations, with one retrospective study comparing antivenom costs for copperhead envenomations showing a significant cost reduction when Fab2AV was used [23]. Based on the results of the current study, there would be a 3.14% difference in acquisition costs of the median total vials of FabAV vs. Fab2AV for suspected *Agkistrodon* envenomations, favoring Fab2AV ($15,010 vs. $14,540.4). In comparing the cases without additional vials, the difference in acquisition costs for total vials would be 64% favoring FabAV ($6,004 vs. $11,309). This difference is even greater when comparing only envenomations due to identified copperheads. In response to a comment [24], authors of the previous study stated “treatment patterns, treatment protocols and clinical choices made at the bedside for antidote administration were all outside the scope” of their study [25]. With a similar number of administered vials between groups, it is unknown what percentage of vials were maintenance doses and, consequently, the cost difference without them. Antivenom cost analyses are influenced by numerous factors. Heterogeneity in initial dosing and use of maintenance dosing, geographic envenomation epidemiology, and wholesale pricings are just a few variables affecting the determination of a cost benefit to stocking one antivenom over another. Our study showed more frequent control of *Agkistrodon* envenomations with FabAV vs. Fab2AV at manufacturer recommended initial dosing. While the post-hoc analysis suggests potential for economic benefit when as-needed dosing, specifically, is employed, generalizability is limited.

### Limitations

There are several limitations to this study potentially impacting the quality of evidence presented. Reviewers were not blinded to the outcome measures or the antivenom within each case, leaving potential for bias with regard to vials being categorized as control or maintenance. Also, the study’s retrospective nature required reliance on provider documentation and medication administration records to designate vials as control or maintenance. No medical toxicologists were practicing in this hospital system during the study period, and emergency department and inpatient providers determined need for antivenom with inconsistent documentation of consultation with the poison center. Variation in practice may have resulted in administration of unnecessary vials and discrepancies in documentation may have contributed to incorrect categorization of vials. Only records from the current hospital system were available for review, and no follow-up or outpatient data were available to determine differences in short or long term morbidity between groups.

Identification of the snake species was dependent on chart review, with reviewers only able to confirm the species with pictures in a handful of cases; however, the proportion of each species is comparable to Louisiana Poison Center data. The current study showed 60% of identified snakes were copperheads and 29% cottonmouths compared to 55% copperheads and 30% cottonmouths from native U.S. crotaline envenomation calls to the poison center between 2004 and 2014. Rattlesnakes were responsible for 15% of those cases involving an identified U.S. crotaline compared to 11% in our study. While impossible to confirm culprit species based on non-expert suppositions or clinical effects alone, none of the included cases in the current study were associated with severe hemotoxicity or neurologic signs to favor envenomation by one of two Louisiana rattlesnakes over envenomation by one of the more common *Agkistrodon* species.

The study design was before-and-after, with FabAV cases occurring first over a specific timeframe followed by Fab2AV cases, with little overlap. There is evidence of intraspecific variation in venom composition in certain crotalines [26]. It is possible that the cases during the Fab2AV period involved more potent venoms, increasing the total vials or likelihood of requiring additional control vials. Other temporal factors including evolution of physician practice could have led to increased antivenom usage during the Fab2AV period.

Finally, the majority of patients in the FabAV cohort received maintenance vials. It is unknown to what degree the additional vials of either antivenom contributed to initial control of venom effects, potentially altering the number of cases requiring additional doses for control. Non-toxicologists are more likely to adhere to FDA recommended dosing, which includes FabAV maintenance vials regardless of species despite benefit being seen only in studies involving primarily rattlesnakes and notably excluding copperheads [10]. This firm adherence to the manufacturer recommended maintenance vials is not consistent with the majority of toxicologists’ management of crotaline envenomations [12], and FabAV maintenance vials have shown to be inconsequential in rates of recurrence of venom effects after initial control of copperhead envenomations [27].

## Conclusion

In this retrospective analysis of suspected *Agkistrodon* envenomations, control of venom effects was established with the initial antivenom dose more frequently in patients who received FabAV compared to those who received Fab2AV. These results suggest potential benefit to hospitals stocking FabAV in Louisiana and possibly other *Agkistrodon-*predominant regions. A randomized controlled trial, or other prospective analysis, comparing efficacy and cost of FabAV to Fab2AV for *Agkistrodon* envenomations is needed.

## Data Availability

Data in this manuscript were previously presented at ACMT’s Annual Scientific Meeting, Vancouver, BC, 2025.
